# Devulcanization of ground tire rubber: microwave and thermomechanical approaches

**DOI:** 10.1038/s41598-020-73543-w

**Published:** 2020-10-06

**Authors:** Dániel Ábel Simon, Dávid Zoltán Pirityi, Tamás Bárány

**Affiliations:** Department of Polymer Engineering, Faculty of Mechanical Engineering, Budapest University of Technology and Economics, Műegyetem rkp. 3., Budapest, 1111 Hungary

**Keywords:** Polymers, Mechanical engineering

## Abstract

We devulcanized ground tire rubber (GTR) in a laboratory microwave oven and an internal mixer, measured the soluble content and the cross-link density of the samples, and then used Horikx’s analysis. The results showed that microwave treatment caused severe degradation of the polymer chains, while in the case of thermomechanical devulcanization, the selective scission of covalent cross-links is more common. Four devulcanized ground tire rubber (dGTR) samples were chosen for further study and three groups of samples were produced: dGTR samples containing vulcanizing agents and different amounts of paraffin oil (dGTR-based mixtures), natural rubber-based rubber mixtures with different dGTR contents and reference rubber mixtures with dGTR-based mixtures (increased vulcanizing agent contents). Cure characteristics showed a plasticizer-like effect of dGTR. Tensile and tear strength decreased drastically with increasing dGTR content; however, elongation at break values did not follow such a trend. Mechanical properties improved with increased vulcanizing agent contents. The examined properties of the samples improved even further with the use of thermomechanically devulcanized samples. Horikx’s analysis showed that this is attributable to moderate polymer chain scission.

## Introduction

The amount of waste rubber products, especially that of waste tires, is rapidly increasing. The traditional methods of waste tire management have been stockpiling or landfilling, both of which are short-term solutions and pose significant health and environmental problems^1^. Therefore, many studies focus on the reclamation and recycling of waste rubber products and tires, but reuse and recycling are a challenge due to the 3D cross-linked structure of rubbers. According to the EU waste hierarchy, the reuse of these products is a favorable solution. However, existing approaches, such as the application of waste tires as safety barriers on racetracks and retreading tires, creates new products of reduced quality and functionality.

Another way to use scrap rubber is to burn it in furnaces or convert it to liquid fuel in pyrolysis reactors in an oxygen-free environment. Tires have a high heat value (ca. 32.6 MJ/kg), which is even higher than that of coal (ca. 18.6–27.9 MJ/kg). The end product of pyrolysis is carbon black and an oil-like material, which can be processed like petroleum^2,3^.

The best way of disposing of waste tires and other rubber products is to turn them into a ground powder (ground tire rubber, GTR)^4^, which can be produced in different ways: mechanical grinding at ambient or cryogenic temperatures^5^, and waterjet milling. In waterjet milling, a high-pressure water beam grinds rubber waste. Compared to mechanical grinding, smaller particles can be obtained, and rubber degradation can also be avoided, though the final material needs to be dried.

GTR can be used as an additive without any physical or chemical treatment in asphalt and can be bound together with polyurethane. GTR can be blended with thermoplastic^4,5^ or thermoset^6–9^ polymers with or without compatibilization^10,11^. However, the volume of these applications cannot meet the ever-increasing need for rubber recycling.

Devulcanization may offer a solution to this problem. This process is suitable to selectively break covalent cross-links in elastomeric materials, keeping the polymer backbone intact. New molecules form, and they can create new bonds on the surface of the GTR particles^4,12–15^, enhancing adhesion between devulcanized GTR (dGTR) particles and other polymers. Consequently, the amount of recycled rubber in new rubber products can be increased without compromising their mechanical properties. Conventional devulcanization techniques, such as thermomechanical^16,17^, thermochemical^18^, mechanochemical^19^, and microwave^12,13,20–23^ devulcanization, have decades-long history of research^24^. There are other devulcanization methods utilizing ultrasound^25,26^, chemolithotrophic bacteria^27^, and supercritical carbon dioxide^28^. Each method has its advantages and disadvantages. Microwave-treated GTR has good properties, and the technique has a promise of high productivity. Microwave devulcanization takes advantage of volumetric heating: a fast and uniform rise in temperature can be achieved. The selective scission of sulfuric cross-links is possible with the right parameters (temperature, exposure time, microwave power). The process does not require additional chemicals and is considered an eco-friendly technology. A major drawback of microwave devulcanization is that nonpolar elastomers like NR, styrene-butadiene rubber (SBR) or ethylene propylene diene monomer rubber (EPDM), have low microwave absorbance. However, carbon black, which is often used as a filler in rubber products^14^, has good microwave absorbance and dissipates its energy in the form of heat^29^.

The effectiveness of devulcanization can be evaluated with the soluble content of the rubber sample. It can be directly measured via Soxhlet extraction, during which a small amount of organic solvent is repeatedly distilled to dissolve the soluble components of a solid material. Many studies, addressing microwave devulcanization, focused on the effect of different exposure times of GTR to microwaves. The accepted conclusion was that the longer the exposure time, the larger the soluble fraction (sol fraction) of the samples^13^. Garcia et al.^20^ devulcanized GTR with microwaves and were able to increase the sol fraction from 14 to 31% after 7 min of treatment. They determined that in addition to the breaking of S–S and C–S bonds, the main chain was also degraded. However, sol fraction on its own is not a sufficient indicator of devulcanization. The cross-link density of dGTR is also an essential factor. The degree of devulcanization can be defined as the percentage decrease in the cross-link density of a sample. De Sousa et al.^10^ found that the higher the amount of energy absorbed during the treatment, the higher the final temperature of the GTR, hence the higher the decrease in cross-link density. The FTIR analysis of dGTR can also reveal the structural changes that take place during devulcanization^13,21^. Therefore, FTIR is a suitable supporting method.

Horikx’s analysis^30^ is a commonly accepted method that establishes a mathematical relationship between the sol fraction and the degree of devulcanization. Ultimately, it can indicate whether the main phenomenon during devulcanization is random scission of the polymer chains or selective scission of the covalent cross-links^31^. Horikx’s analysis is becoming a widely used method to grade devulcanization. There are many examples of its use in scientific literature: assessing microwave devulcanization of recycled NR^12,32^, thermomechanical devulcanization of GTR^33^, thermochemical devulcanization of NR and EPDM^34^ and microbiological devulcanization of NR^35^.

In our previous article, we showed the effects of different GTRs (mechanically ground and water-jet milled) with different particle sizes on the devulcanization process and optimized the parameters of microwave devulcanization^36^. Furthermore, we added dGTR to polypropylene-based thermoplastic dynamic vulcanizates (TDV) to verify our method as a means of GTR recycling. We found that virgin rubber can be replaced with up to 20 wt% devulcanizate without compromising the mechanical properties of the resulting TDV^37^.

In this study, we compared the applicability of microwave-devulcanized and mechanically devulcanized GTR in virgin NR. First, we selected the ideal devulcanizates for further study based on Horikx’s analysis. We produced NR-based mixtures incorporating various amounts of the four selected dGTR samples. Finally, we tested the mechanical and physical properties of the resulting rubber samples to determine the effectiveness of our recycling processes.

## Experimental

### Materials

Waterjet-milled crumb rubber was provided by Aquajet Ltd. (Budapest, Hungary). The material originated from the tread area of truck tires. Therefore, this type of GTR is a high-purity material. According to TGA measurements, it contains 50–55 phr of NR, 45–50 phr of synthetic rubber, 4–6 phr of oil, 33–37 phr of carbon black, and 7.5 phr of residual additives. We chose a general-purpose natural rubber for our investigations. Table 1 contains the manufacturers, types, and basic properties of GTR and NR.

**Table 1:** Types and producers of raw materials.

Abbreviation	GTR	NR
Manufacturer, type	Aquajet Ltd., Budapest, Hungary	NR TSR 10 Sud Comoe Caoutchuc, Aboisso, Ivory Coast
Main properties	Waterjet-milled truck tire tread, particle size between 200 and 400 μm	Mooney viscosity (ML, 1 + 4, 100 °C): 55–65

The additives of rubber mixtures and their suppliers were the following: zinc oxide (ZnO, S.C. Werco Metal S.r.l., Zlatna, Romania), stearic acid (Oleon, Ertvelde, Belgium), N772 carbon black (Omsk Carbon Group OOO, Omsk, Russian Federation), paraffin oil (Ipol Lubricants, Mumbai, India), tetramethyl thiuram disulfide (TMTD, Akrochem Corporation, Akron, Ohio, USA), N-cyclohexyl-2-benzothiazole sulfonamide (CBS, Rhein Chemie, Mannheim, Germany) and sulfur (Ningbo Actmix Rubber Chemicals Co., Ltd., Ningbo, China). The particle size distribution of GTR was published in our previous paper^36^.

### Devulcanization of GTR

Microwave devulcanization of GTR was carried out in a BP-125/50 type laboratory microwave oven, produced by Microwave Research Inc. (Carol Stream, Illinois, USA). We heated 50 and 100 g batches of GTR up to 200 °C with a heating rate of 6 °C/min. The microwave power was controlled by a PID controller that used data from a thermocouple that was continuously measuring the temperature of the rubber inside the oven. A motorized stirring system was installed to the microwave oven to insure homogeneous temperature. After the temperature reached 200 °C, the material was taken out and allowed to cool to room temperature. In some cases, the samples were heat-treated at 140 °C in a Venticell LSIS-B2V (MMM Group, Monroe, Louisiana, USA) laboratory oven prior to devulcanization. The parameters of microwave devulcanization, and the nomenclature of the samples can be seen in Table 2.

**Table 2:** Parameters of microwave devulcanization and the abbreviation of the samples.

Microwave devulcanization			
Abbreviations	Batch size (g)	Achieved temperature (°C)	Heat treatment before devulcanization (h)
dGTR_MW_50g	50	200	–
dGTR_MW_50g_1	50	200	1
dGTR_MW_50g_2	50	200	2
dGTR_MW_100g	100	200	–
dGTR_MW_100g_1	100	200	1
dGTR_MW_100g_2	100	200	2
GTR_H_2	100	140	2 (heat treatment only)

Thermomechanical devulcanization was performed in a Brabender Plasti-corder internal mixer (Brabender GmbH & Co., Duisburg, Germany). The duration of the treatment was 10 min, and the chamber volume was 50 cm^3^. The parameters of thermomechanical devulcanization and the abbreviations of the samples are listed in Table 3. The GTR was kept at ambient conditions before treatment.

**Table 3:** Parameters of thermomechanical devulcanization and the abbreviation of the samples.

Thermomechanical devulcanization		
Abbreviations	Rotor speed (rpm)	Achieved temperature (°C)
dGTR_TM_40/160	40	160
dGTR_TM_40/170	40	170
dGTR_TM_40/180	40	180
dGTR_TM_40/190	40	190
dGTR_TM_40/200	40	200
dGTR_TM_120/160	120	160
dGTR_TM_120/170	120	170
dGTR_TM_120/180	120	180
dGTR_TM_120/190	120	190
dGTR_TM_120/200	120	200

### Characterization of GTR and dGTR

GTR and dGTR were characterized by Soxhlet extraction in toluene, according to Eq. (1). The insoluble fraction, or gel fraction of the rubber can be separated from the soluble fraction with this extraction technique. High sol content of a devulcanizate is a good indicator of its processability. It indicates the presence of small polymer molecules ready to be reintegrated into the rubber matrix via curing. These molecules can be effectively separated via Soxhlet extraction. We ran the extraction for 18 h and then dried the samples for 12 h at 80 °C to remove the solvent. We weighed each sample twice: before extraction and after drying.

1 $$Sol \;\;Fraction \; \left({\%}\right)=\left(1-\frac{{M}_{f}}{{M}_{i}}\right) \cdot 100$$

where *M*_*i*_ and *M*_*f*_ stand for the mass of rubber before and after the extraction, respectively.

The cross-link density of untreated GTR and devulcanizates was determined via swelling tests according to ASTM D 297-15. We calculated the cross-link density values using the Flory-Rehner Eq. (2)^38^ after equilibrium swelling (72 h followed by drying to constant mass at 80 °C) in toluene.

2 $${\nu }_{e}=\frac{-[\mathrm{ln}\left(1-{V}_{r}\right)+{V}_{r}+{\chi }_{1} \cdot {{V}_{r}}^{2}]}{[{V}_{1} \cdot ({{V}_{r}}^\frac{1}{3}-{V}_{r})/ 2]}$$

where ${\nu }_{e}$ is cross-link density (mol/cm^3^); *V*_*1*_ is the molar volume of the solvent (for toluene: 106.13 cm^3^/mol); ${\chi }_{1}$ is the rubber-solvent interaction parameter (0.39), and *Vr* means the volume fraction of rubber in the swollen sample, which can be determined with the Ellis and Welding Eq. (3)^31^.

3 $${V}_{r}=\frac{\frac{{m}_{r}}{{\rho }_{r}}}{\frac{{m}_{r}}{{\rho }_{r}}+\frac{{m}_{s}}{{\rho }_{s}}}$$

where *m*_*s*_ is the mass of the swollen rubber sample (g), *m*_*r*_ is the mass of the dry rubber sample (g), ${\rho }_{s}$ is the density of the solvent, toluene (0.8669 g/cm^3^) and ${\rho }_{r}$ is the density of the rubber sample (1.20 g/cm^3^).

The degree of devulcanization was calculated with Eq. (4)^12^

4 $$Dev \left(\mathrm{\%}\right)=\left(1-\frac{{\upnu }_{\mathrm{f}}}{{\upnu }_{\mathrm{i}}}\right) \cdot 100$$

where ${\nu }_{f}$ is the cross-link density of the devulcanized sample and ${\nu }_{i}$ is the cross-link density of untreated GTR.

### Formulation and preparation of rubbers containing GTR and dGTR

After the evaluation of the devulcanization experiments, we selected four types of dGTR (dGTR_MW_100g_2, dGTR_TM_40/160, dGTR_TM_40/200 and dGTR_TM_120/200) and added vulcanizing agents to them with an internal mixer. The dGTR samples were chosen based on the results of Horikx’s analysis. We investigated the effects of different amounts of vulcanizing agents and paraffin oil. The formulations of rubber mixtures are shown in Table 4.

**Table 4:** The dGTR-based compounds and their abbreviations (values in phr).

Abbreviation	dGTR_MW_100g_2	dGTR_TM_40/160	dGTR_TM_40/200	dGTR_TM_120/200	ZnO	Stearic acid	Paraffin oil	CBS	TMTD	Sulfur
dGTR_MW_100g_2_A	100	0	0	0	10	2	20	1.25	0.6	0.6
dGTR_MW_100g_2 0.5A	100	0	0	0	5	1	10	0.625	0.3	0.3
dGTR_MW_100g_2_B	100	0	0	0	10	2	10	1.25	0.6	0.6
dGTR_MW_100g_2_0.5B	100	0	0	0	5	1	5	0.625	0.3	0.3
dGTR_TM_40/160_A	0	100	0	0	10	2	20	1.25	0.6	0.6
dGTR_TM_40/200_A	0	0	100	0	10	2	20	1.25	0.6	0.6
dGTR_TM_120/200_A	0	0	0	100	10	2	20	1.25	0.6	0.6

To assess the usability of dGTR in rubbers, we added different amounts of dGTR and GTR (as reference) to NR-based compounds. The recipes of the rubber compounds are shown in Table 5. We introduced a simplified notation: MW denotes the dGTR_MW_100g_2 microwave-devulcanized sample.

**Table 5:** The NR-based rubber compounds containing microwave-devulcanized ground tire rubber and their abbreviations (values in phr).

Abbreviation	NR	ZnO	Stearic acid	Carbon black (N 772)	dGTR_MW_100g_2	dGTR_MW_100g_2_0.5A	dGTR_MW_100g_2_A	dGTR_MW_100g_2_0.5B	dGTR_MW_100g_2_B	GTR	Paraffin oil	CBS	TMTD	Sulfur
NR_REF	100	10	2	60	0	0	0	0	0	0	10	1.25	0.6	0.6
NR_REF_WO	100	10	2	60	0	0	0	0	0	0	0	1.25	0.6	0.6
NR_REF_WO_dGTR_MW	100	10	2	60	10	0	0	0	0	0	0	1.25	0.6	0.6
NR_dGTR_MW_50	100	10	2	60	50	0	0	0	0	0	10	1.25	0.6	0.6
NR_dGTR_MW_100	100	10	2	60	100	0	0	0	0	0	10	1.25	0.6	0.6
NR_dGTR_MW_185	100	10	2	60	185	0	0	0	0	0	10	1.25	0.6	0.6
NR_dGTR_MW_100_0.5A	100	10	2	60	0	100	0	0	0	0	10	1.25	0.6	0.6
NR_dGTR_MW_100_A	100	10	2	60	0	0	100	0	0	0	10	1.25	0.6	0.6
NR_dGTR_MW_50_0.5B	100	10	2	60	0	0	0	50	0	0	10	1.25	0.6	0.6
NR_dGTR_MW_50_B	100	10	2	60	0	0	0	0	50	0	10	1.25	0.6	0.6
NR_dGTR_MW_100_0.5B	100	10	2	60	0	0	0	100	0	0	10	1.25	0.6	0.6
NR_dGTR _MW_100_B	100	10	2	60	0	0	0	0	100	0	10	1.25	0.6	0.6
NR_GTR_100	100	10	2	60	0	0	0	0	0	100	10	1.25	0.6	0.6

We prepared a reference sample (NR_REF), a reference sample without paraffin oil (NR_REF_WO), and a reference sample where paraffin oil was replaced with dGTR_MW_100g_2 (NR_REF_WO_dGTR_MW). In the abbreviation of the other samples, the number (50, 100, or 185) means the dGTR content in parts per hundred rubber (phr). In the case of samples ending with "A" or "B", mixing consisted of two steps. In the first step, the dGTR was compounded with vulcanizing agents according to Table 4. Then this untreated dGTR mixture was added to the original rubber mixture. Table 6 shows rubber mixtures containing thermomechanically devulcanized GTR. In summary, the dGTR_MW_100g_2 microwave-devulcanized and the dGTR_TM_40/160, dGTR_TM_40/200, dGTR_TM_120/200 thermomechanically devulcanized samples were incorporated in the rubber mixtures.

**Table 6:** The NR-based rubber compounds containing termomechanically devulcanized ground tire rubber and their abbreviations (values in phr).

Abbreviation	NR	ZnO	Stearic acid	Carbon black (N 772)	dGTR_TM_40/160	dGTR_TM_40/200	dGTR_TM_120/200	dGTR_TM_40/160_A	dGTR_TM_40/200_A	dGTR_TM_120/200_A	Paraffin oil	CBS	TMTD	Sulfur
NR_dGTR_TM_40/160_100	100	10	2	60	100	0	0	0	0	0	10	1.25	0.6	0.6
NR_dGTR_TM_40/200_100	100	10	2	60	0	100	0	0	0	0	10	1.25	0.6	0.6
NR_dGTR_TM_120/200_100	100	10	2	60	0	0	100	0	0	0	10	1.25	0.6	0.6
NR_dGTR_TM_40/160_100_A	100	10	2	60	0	0	0	100	0	0	10	1.25	0.6	0.6
NR_dGTR_TM_40/200_100_A	100	10	2	60	0	0	0	0	100	0	10	1.25	0.6	0.6
NR_dGTR_TM_120/200_100_A	100	10	2	60	0	0	0	0	0	100	10	1.25	0.6	0.6

The rubber ingredients were mixed in a Brabender Plasti-corder internal mixer at 50 °C and 40 rpm. The order of appearance for the components in Tables 4, 5, 6 (left to right) also reflects the order of mixing. The compounds were vulcanized with a Teach-Line Platen Press 200E (Dr. Collin GmbH, Munich, Germany) hot press. The pressure applied was 2.8 MPa, and the temperature was 160 °C. Each compound was cured for t_90_ (time necessary to reach 90% vulcanization). These time values were obtained from separate rheometer measurements.

### Characterization of the rubber mixture and cured rubber sheets

The curing curves of the rubber compounds were recorded with a MonTech Monsanto R100S rheometer (MonTech Werkstoffprüfmaschinen GmbH, Buchen, Germany) in isothermal (T = 160 °C) time sweep mode (1.667 Hz, 1° angle) for 30 min.

Hardness was tested according to the ISO 48-4:2018 Shore A method on a Zwick H04.3150.000 hardness tester (Zwick GMBH., Ulm, Germany) on the cured rubber sheets. Ten tests were performed on each compound, followed by the calculation of the average and standard deviation values.

The tensile mechanical properties of the compounds were investigated according to the ISO 37:2017 standard on a Zwick Z250 universal testing machine with a 20 kN load cell (Zwick GmbH, Ulm, Germany). Type 1 specimens with a clamping length of 60 mm were loaded at a crosshead speed of 500 mm/min. Tear tests were performed on the same testing machine, and test speed was according to the ISO 34-1:2015 standard (Type C specimen), with a clamping length of 56 mm. Both tests were run at room temperature. The average and standard deviation of the tensile strength, tear strength, and elongation at break values were calculated based on five tests for each compound.

## Results and discussion

### Devulcanization of GTR

Table 7 lists the sol content, cross-link density, and the degree of devulcanization of the microwave-devulcanized samples. First, we treated 50 g batches of GTR and observed an increase in sol fraction and a decrease in cross-link density, indicating devulcanization. Later, we scaled up to batch sizes of 100 g to improve productivity. The sol content remained unchanged. The power of the microwave oven was enough to heat the GTR to 200 °C without the need to increase the duration of the treatment. A further increase in batch size was not possible because of the size of the instrument.

**Table 7:** The sol fraction of GTR after microwave devulcanization.

Sample	Sol fraction (%)	Cross-link density (10^–4^ mol/cm^3^)	Devulcanization (%)
GTR	10.0 ± 1.2	19.4 ± 0.7	–
dGTR_MW_50g	16.1 ± 0.8	12.0 ± 0.6	38.1
dGTR_MW_50g_1	17.0 ± 0.6	11.9 ± 0.4	38.7
dGTR_MW_50g_2	25.1 ± 1.1	8.5 ± 0.6	56.2
dGTR_MW_100g	15.8 ± 0.7	12.2 ± 0.5	37.1
dGTR_MW_100g_1	17.3 ± 0.9	12.5 ± 0.4	35.6
dGTR_MW_100g_2	28.5 ± 1.3	8.2 ± 0.7	57.7
GTR_H_2	20.4 ± 0.4	11.0 ± 0.8	43.3

An hour-long heat treatment (at 140 °C) before devulcanization did not cause a significant change in the sol content. However, when the samples were treated for two hours, the sol fraction increased significantly. The degree of devulcanization followed a similar trend. In other words, the sol content increased significantly, while the cross-link density did not decrease considerably. That indicates the degradation of the polymer chains. Similar conclusions can be drawn for the GTR_H_2 sample.

Table 8 shows the sol content, cross-link density, and the degree of devulcanization of the samples after thermomechanical devulcanization. The trends are clear; increasing temperature and rotor speed lead to increasing sol content. At the same time, there is a continuous decrease in cross-link density.

**Table 8:** The sol fraction of GTR after thermomechanical devulcanization.

Sample	Sol fraction (%)	Cross-link density (10^–4^ mol/cm^3^)	Devulcanization (%)
GTR	10.0 ± 1.2	19.4 ± 0.7	–
dGTR_TM_40/160	14.4 ± 0.7	7.3 ± 0.5	62.4
dGTR_TM_40/170	18.0 ± 0.5	6.8 ± 0.4	65.0
dGTR_TM_40/180	19.1 ± 0.6	6.3 ± 0.4	67.5
dGTR_TM_40/190	20.3 ± 0.9	5.9 ± 0.3	69.6
dGTR_TM_40/200	25.8 ± 0.7	4.0 ± 0.6	79.4
dGTR_TM_120/160	16.3 ± 0.6	6.9 ± 0.5	63.4
dGTR_TM_120/170	21.6 ± 0.8	7.1 ± 0.4	65.2
dGTR_TM_120/180	23.4 ± 0.6	6.3 ± 0.6	67.5
dGTR_TM_120/190	25.1 ± 0.5	3.8 ± 0.5	80.4
dGTR_TM_120/200	30.2 ± 0.7	3.7 ± 0.3	80.9

**Figure 1:**
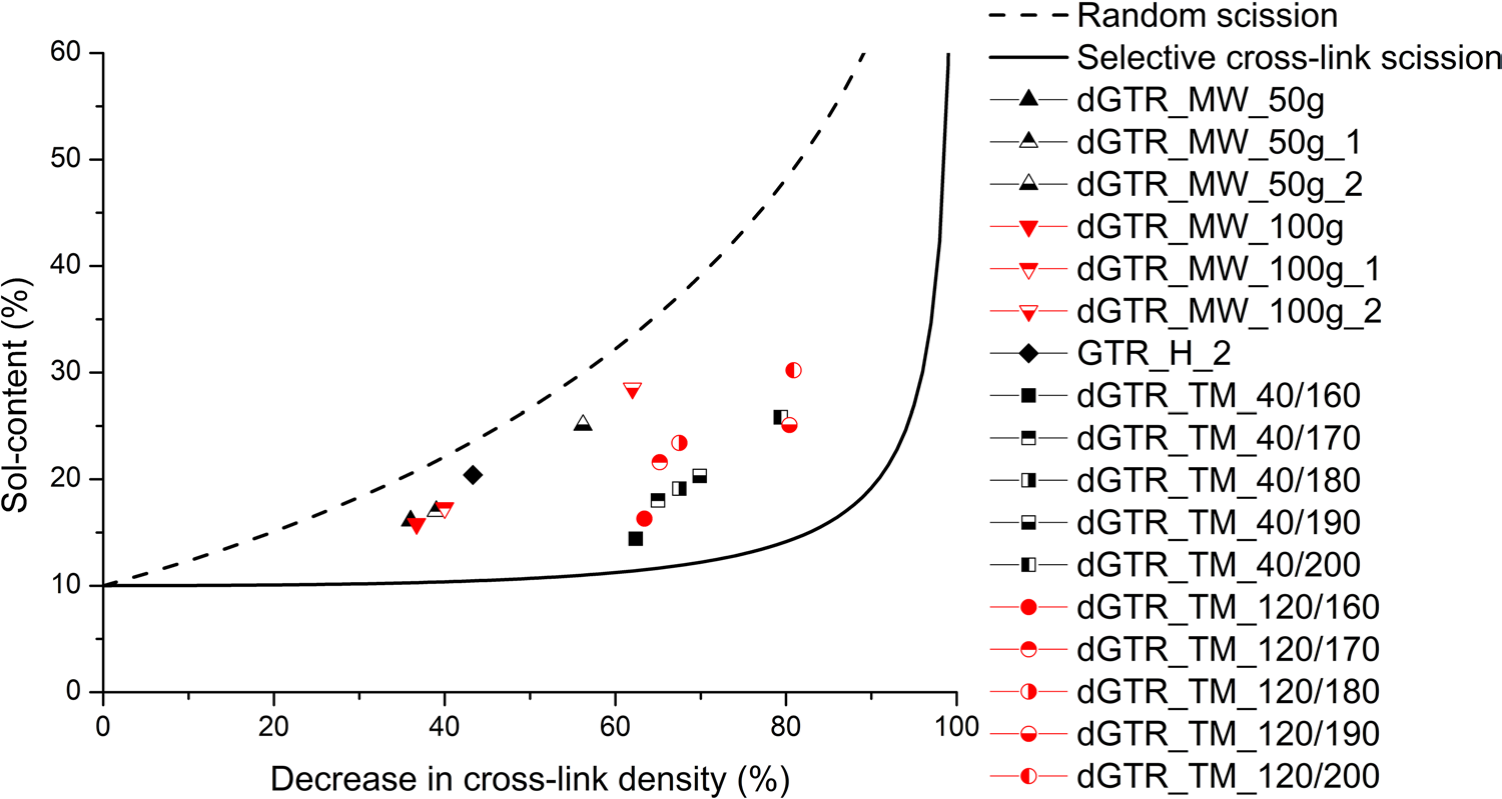
Horikx's plot, sol content of devulcanized samples versus decrease in the cross-link density.

We used Horikx’s analysis to determine the relationship between the sol fraction after the degradation of the 3D cross-link structure of rubber and the relative decrease in cross-link density. Horikx derived an extensive method to identify and illustrate whether the degradation of a polymer is dominated by random chain scission or the selective breakdown of cross-links (i.e. devulcanization). He identified two different scenarios: random chain scission and scission of the cross-links. In the case of main chain scission, Eq. (5) shows the relationship between the soluble fraction of the polymer and the relative decrease in the number of elastically active network chains.

5 $$1-\frac{{v}_{f}}{{v}_{i}}=1-\frac{{\left(1-\sqrt{{s}_{f}}\right)}^{2}}{{\left(1-\sqrt{{s}_{i}}\right)}^{2}}$$

where ${v}_{i}$ stands for the initial cross-link density, ${v}_{f}$ stands for cross-link density after degradation, ${s}_{i}$ stands for the initial sol fraction of the polymer and ${s}_{f}$ stands for the sol fraction of the polymer after treatment^30,34^. Based on Eq. (5) a curve of random scission can be plotted (Fig. 1).

The second scenario involves only cross-link cleavage, with no degradation of the polymer main chain. Equation (6) was formulated based on Horikx’s research^30^.

6 $$1-\frac{{v}_{f}}{{v}_{i}}=1-\frac{{\gamma }_{f}{\left(1-\sqrt{{s}_{f}}\right)}^{2}}{{\gamma }_{i}{\left(1-\sqrt{{s}_{i}}\right)}^{2}}$$

where ${\gamma }_{i}$ and ${\gamma }_{f}$ stand for the initial and final cross-linking index, respectively. The cross-linking index tells the average number of cross-link bonds per polymer chain^30,34^ and it can be determined by Eq. (7)^30^. This curve contributes to selective cross-link scission (Fig. 1).

7 $${\gamma }_{x}={v}_{x}\frac{{M}_{n}}{\rho }$$

where ${\gamma }_{x}$ (−) is the cross-linking index, ${v}_{x}$ (mol/cm^3^) is the cross-link density, M_n_ (g/mol) stands for the number-avarege molecular weight of the rubber and $\rho$ is the rubber density.

In this paper, the initial cross-linking index is approximated by Eq. (8)^39^.

8 $$Sol\;\;Fraction \;(\%)=\frac{\left(2+{\gamma }_{i}\right)-\sqrt{{\gamma }_{i}^{2}+4{\gamma }_{i}}}{2{\gamma }_{i}}$$

Based on Eqs. (5) and (6), the relationship between the sol content and the decrease in cross-link density can be plotted (Fig. 1). The two curves represent the scission of the main chain and the selective degradation of the cross-links. Experimental data can be plotted on the graph, and depending on which curve a data point is closer to, it is possible to infer what is the main phenomenon that occurs during the devulcanization process.

dGTR_MW_50g_2 and dGTR_MW_100g_2 samples had high sol contents, coupled with high crosslink density values. It signified a low degree of devulcanization and suggested the degradation of the polymer chains, as shown in Fig. 1, since the corresponding data points are closer to the random scission curve than the selective cross-link scission curve (Table 9). Thermomechanically devulcanized GTR samples showed more promising results as their data points are located closer to the cross-link scission curve of the Horikx's plot.

**Table 9:** Vertical distance of experimental data points from the selective cross-link scission curve.

Sample	The vertical distance from the selective scission curve (%)
dGTR_MW_50g	53.8
dGTR_MW_50g_1	59.7
dGTR_MW_50g_2	75.0
dGTR_MW_100g	51.9
dGTR_MW_100g_1	67.2
dGTR_MW_100g_2	88.3
GTR_H_2	77.0
dGTR_TM_40/160	13.5
dGTR_TM_40/170	26.8
dGTR_TM_40/180	28.5
dGTR_TM_40/190	30.6
dGTR_TM_40/200	35.4
dGTR_TM_120/160	20.0
dGTR_TM_120/170	44.3
dGTR_TM_120/180	45.5
dGTR_TM_120/190	31.5
dGTR_TM_120/200	45.4

Table 9 shows the vertical distance (in percentage) of experimental data points from the selective cross-link scission curve. If the value is zero, then the data point is on the selective cross-link scission curve. Based on these results, the best sample is dGTR_TM_40/160, and the worst is dGTR_MW_100g_2. But we need to evaluate these results with the degree of devulcanization to get a complete overview. If we look at these two samples, it is easy to determine which method is better, because both samples have almost the same cross-link density; they significantly differ only in sol content. We chose four samples for further study: dGTR_MW_100g_2, dGTR_TM_40/160, dGTR_TM_40/200 and dGTR_TM_120/200.

### Cure characteristics of the rubber compounds

**Figure 2:**
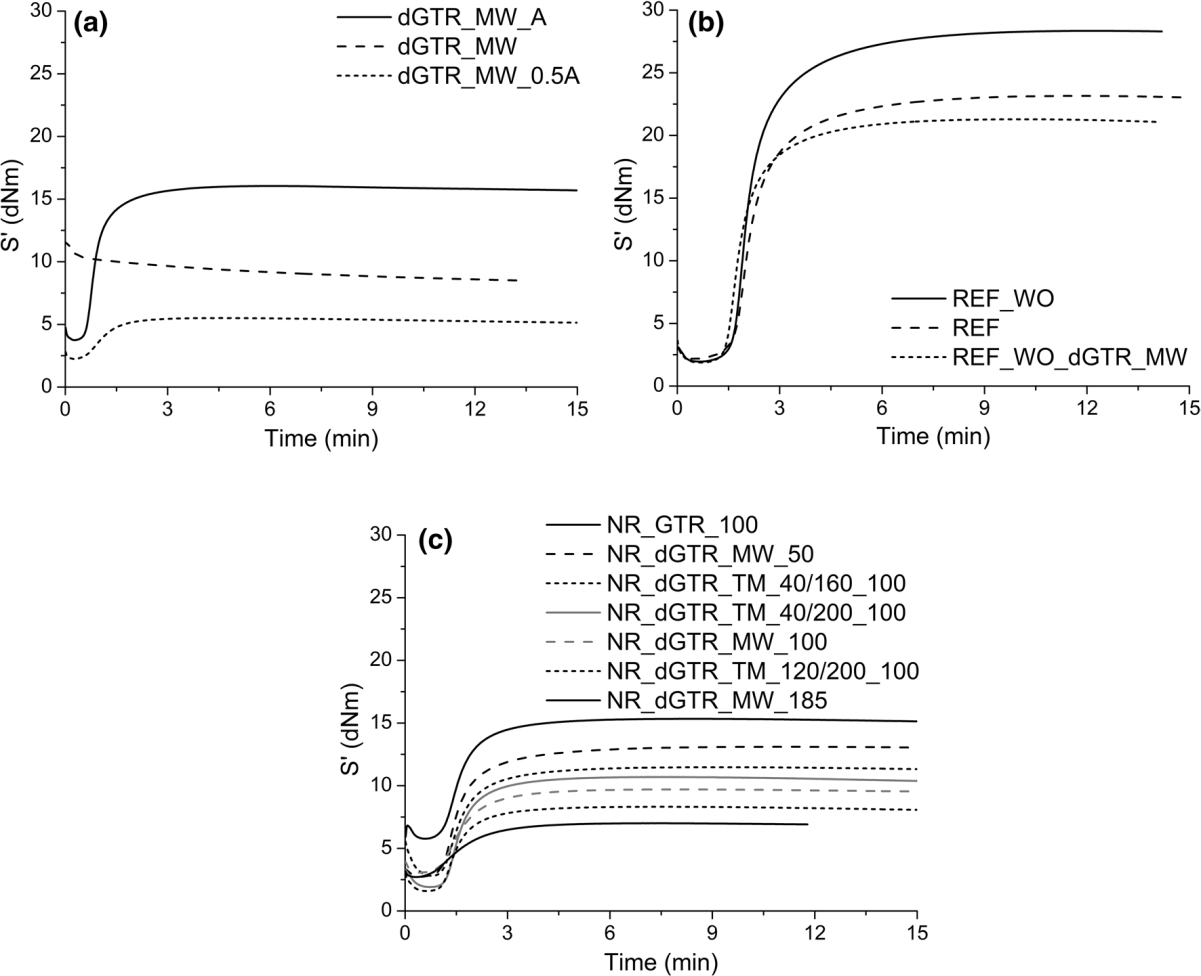
Vulcanization curves of the samples, dGTR and dGTR-based mixtures (**a**), reference samples (**b**) and rubber mixtures containing dGTR (**c**).

**Figure 3:**
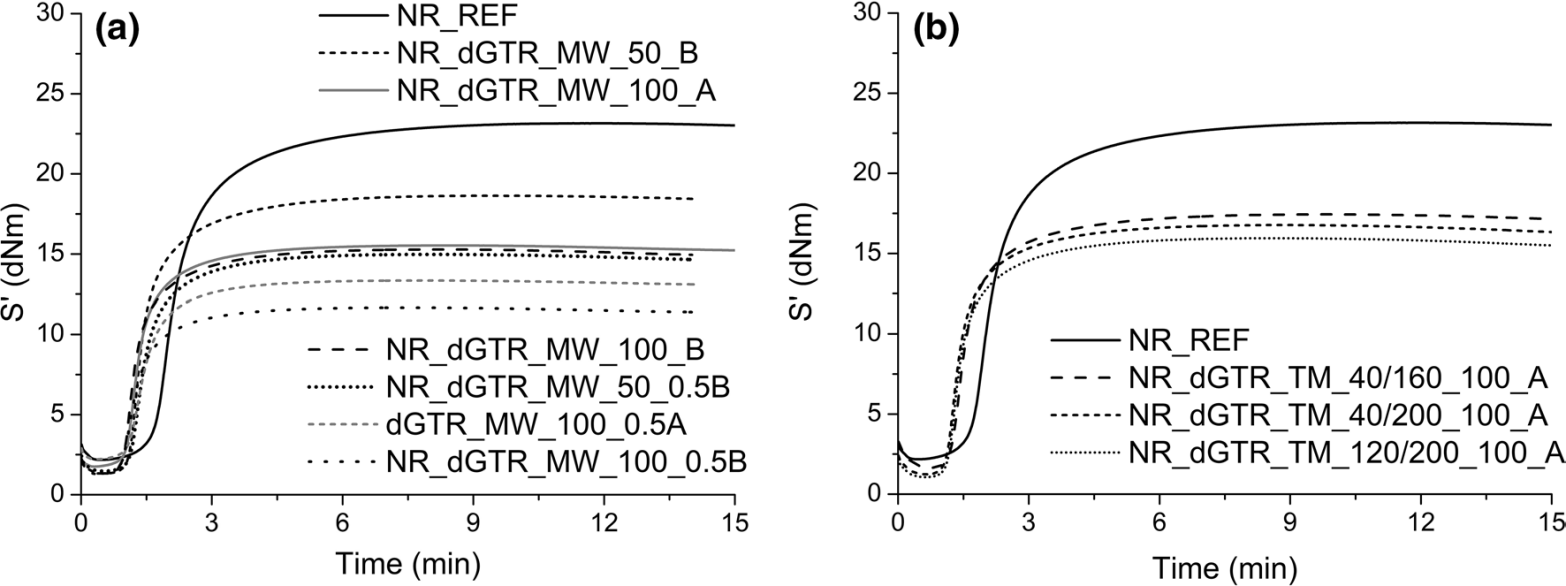
Vulcanization curves of the samples containing microwave devulcanized GTR and extra vulcanizing agents (**a**), samples containing thermomechanically devulcanized GTR and containing extra vulcanizing agents (**b**).

Figures 2 and 3 show the recorded vulcanization curves of the samples. Table 10 shows the cure characteristics of the dGTR-based rubber mixtures.

**Table 10:** Cure characteristics and hardness of the samples.

Sample	t_90_ (min)	t_s2_/t_10_ (min)	Sʹ_min_ (dNm)	Sʹ_max_ (dNm)	Shore A hardness (–)
NR_REF	4.2	1.7	2.2	23.2	57.3 ± 0.7
NR_REF_WO	4.2	1.7	2.0	28.3	58.8 ± 0.9
NR_REF_WO_dGTR_MW	3.5	1.5	1.9	21.3	55.1 ± 0.6
NR_dGTR_MW_50	3.2	1.1	2.7	13.1	51.4 ± 0.3
NR_dGTR_MW_100	3.0	1.1	3.0	9.7	47.9 ± 0.5
NR_dGTR_MW_185	3.2	0.8	2.7	7.0	46.0 ± 0.7
NR_dGTR_TM_40/160_100	3.0	1.2	2.8	11.5	49.5 ± 0.7
NR_dGTR_TM_40/200_100	2.8	1.2	1.9	10.7	47.2 ± 0.4
NR_dGTR_TM_120/200_100	2.7	1.1	1.6	8.3	44.3 ± 0.5
NR_GTR_100	2.9	1.1	5.8	15.3	52.9 ± 0.4
NR_dGTR_MW_100_0.5A	2.6	1.1	2.2	13.3	48.9 ± 0.3
NR_dGTR_MW_100_A	2.6	1.1	1.8	15.6	51.8 ± 0.8
NR_dGTR_MW_50_0.5B	2.7	1.1	1.5	15.0	45.4 ± 1.2
NR_dGTR_MW_50_B	3.0	1.1	1.3	18.6	48.2 ± 1.0
NR_dGTR_MW_100_0.5B	2.4	1.0	1.3	11.7	47.3 ± 0.5
NR_dGTR _MW_100_B	2.6	1.0	1.3	15.3	47.4 ± 1.2
NR_dGTR_TM_40/160_100_A	3.1	1.2	1.6	17.4	51.6 ± 0.6
NR_dGTR_TM_40/200_100_A	2.9	1.1	1.2	16.7	51.1 ± 0.7
NR_dGTR_TM_120/200_100_A	2.9	1.1	1.0	15.9	50.3 ± 0.5
NR_dGTR_MW_100g_2	–	–	–	–	–
NR_dGTR_MW_100g_2_0.5A	2.0	0.6	2.2	5.5	47.3 ± 1.1
NR_dGTR_MW_100g_2_A	1.9	0.7	3.7	16.0	53.7 ± 0.9

First, we tried to revulcanize neat dGTR (dGTR_MW_100g_2 sample) without any vulcanizing agents, but curing did not occur (Fig. 2a). The recorded torque values (S′) showed a continuous decrease with time. It can be inferred that the microwave treatment removed all active sulfur from the sample, inhibiting the formation of new cross-links. During devulcanization, sulfur-based cross-link bonds break. There are very few active sulfur atoms that can take part in the vulcanization process later. The sulfur atoms stay in the system in an inactive form or exit from it, generating sulfur dioxide or hydrogen sulfide. dGTR_MW_100g_2_0.5A and dGTR_MW_100g_2_A samples, containing additional curing agents according to Table 4, vulcanized like conventional rubber, and we were able to determine the main characteristics of vulcanization (Table 10). With devulcanization, active molecules were generated, capable of creating new bonds, therefore cross-links formed during vulcanization with the help of sulfur. We were able to produce a solid homogeneous rubber sheet via hot pressing. S′_max_ values increased because of the extra vulcanizing agents added to dGTR but there is no significant effect of the oil content in dGTR mixtures (A and B samples). Traditional vulcanization curves were recorded in the case of all samples containing vulcanizing agents.

In the case of the NR_REF samples (Fig. 2b), paraffin oil and dGTR had similar plasticizing effects, and dGTR also accelerated curing. For the other samples (Fig. 2c), the trend is clear: S′_max_ values decreased with increasing dGTR content. dGTR has a strong plasticizing effect on the mixtures. Vulcanization time also decreased with dGTR, but independently of its amount. The S′_max_ values also decreased in GTR100 samples, but not as much as in the samples containing 100 phr of dGTR. The S′_min_ values were almost twice as high as in the case of the other mixtures. This behavior is the result of the presence of hard GTR particles, and hence we can observe that dGTR has a stronger plasticizing effect than GTR. In the case of the tested samples with thermomechanically devulcanized rubber content, the lower their cross-link densities were, the lower the respective maximum torque values were (Fig. 2c).

Figure 3 shows the vulcanization curves of NR samples containing microwave devulcanized GTR with extra vulcanization agents (Fig. 3a) and NR samples containing thermomechanically devulcanized GTRs with extra vulcanization agents (Fig. 3b) (samples ending with “A” or “B”). The S′_max_ values increased because of the extra vulcanizing agents added to dGTR; the extra vulcanizing agents increased the number of cross-links in the samples during curing.

Table 10 also shows the hardness of the samples; both GTR and dGTR content decreased the hardness of the compounds.

### Mechanical properties of the cured rubber compounds

We were able to perform tensile tests on samples dGTR_MW_100g_2_A (tensile strength: 2.3 ± 0.3 MPa, elongation at break: 85 ± 12%) and dGTR_MW_100g_2_0.5A (tensile strength: 2.1 ± 0.2 MPa, elongation at break: 78 ± 10%). Even though hot pressing yielded homogeneous, rubber-like sheets, their mechanical properties were quite poor. It is necessary to combine NR with dGTR (the applied curing systems can be seen in Table 4).

**Figure 4:**
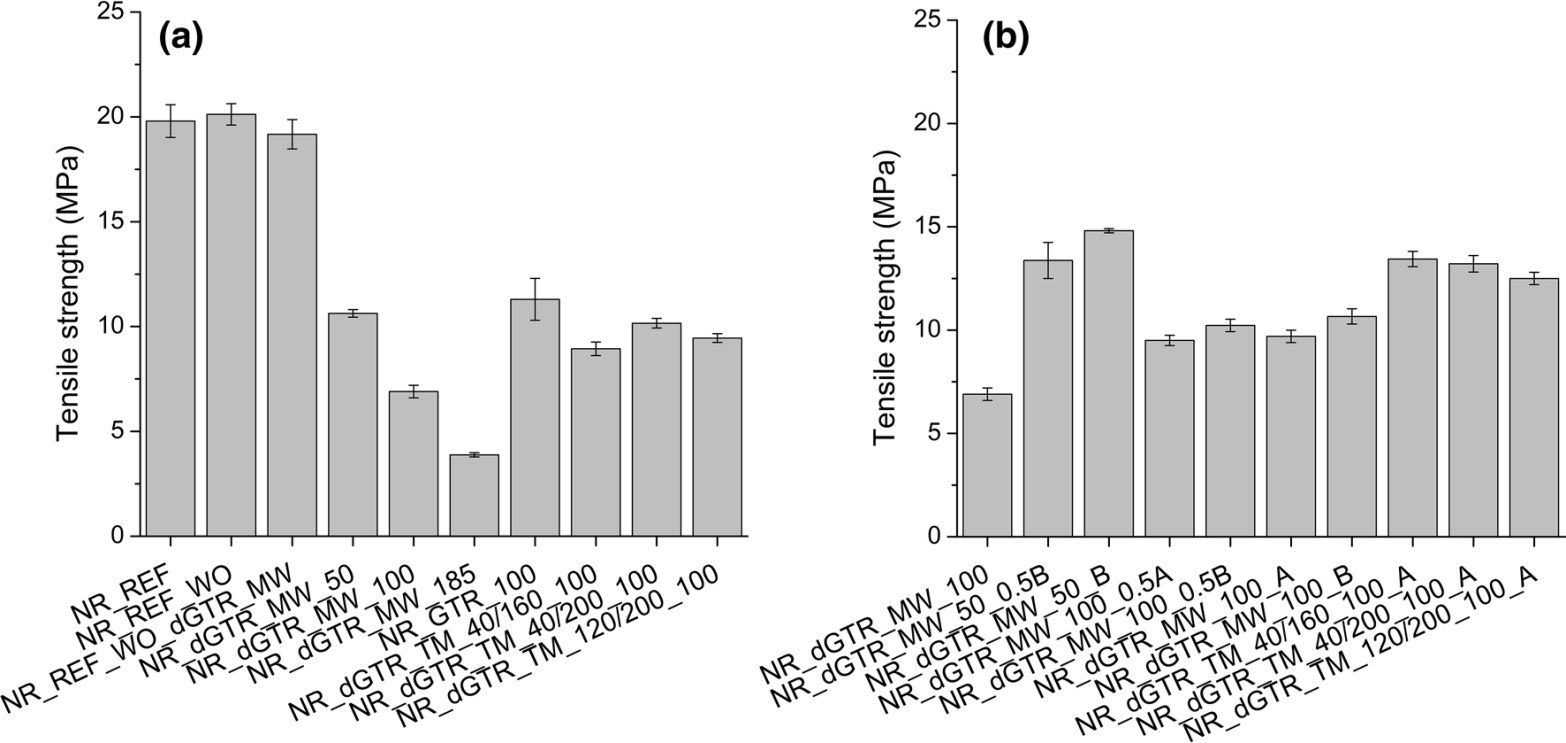
The tensile strength of the samples: mixtures containing dGTR (**a**) and containing dGTR and extra vulcanizing agents (**b**).

Figure 4a shows the tensile strength of the NR-based samples containing different amounts of dGTR. There is no significant difference in tensile strength in the NR_REF samples; paraffin oil and dGTR have a similar effect. dGTR significantly reduced the tensile strength of the samples (samples containing 50, 100, and 185 phr of dGTR_MW_100g_2). The tensile strength values of samples containing thermomechanically devulcanized GTR are higher than those of the samples containing microwave-devulcanized GTR. Based on Horikx’s analysis, the polymer backbone of thermomechanically devulcanized GTRs suffered less severe degradation than that of microwave-devulcanized GTR. Additional vulcanizing agents in dGTR helped recover tensile strength because of the more significant number of cross-links generated compared with other samples (Fig. 4b).

**Figure 5:**
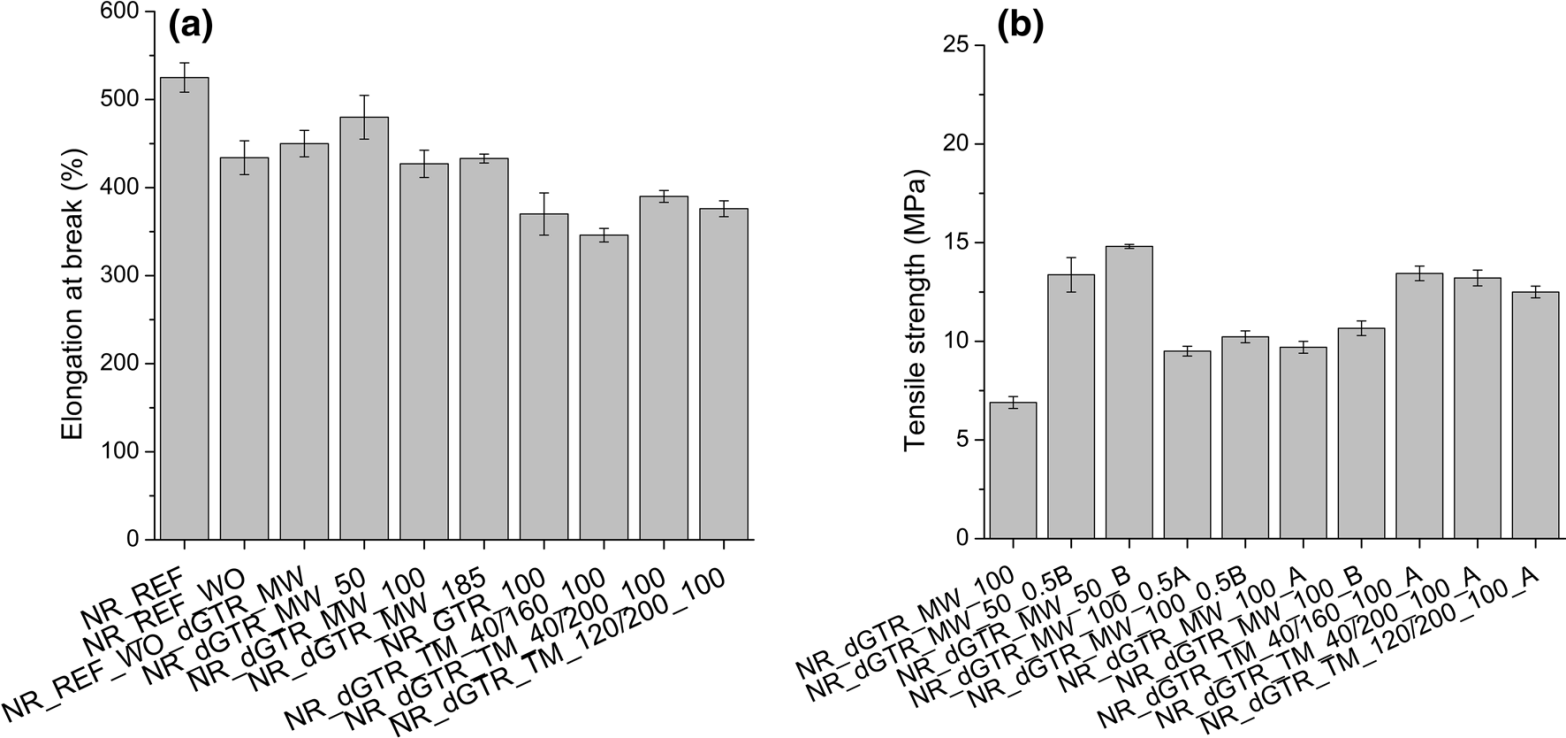
Elongation at break of the samples: mixtures containing dGTR (**a**) and containing dGTR and extra vulcanizing agents (**b**).

The elongation at break of the samples (Fig. 5) decreased slightly when dGTR was used because of the intense plasticizing effect of the GTR particles softened by devulcanization. The tensile strength of the mixture containing GTR (NR_GTR_100) did not decrease as much as that of the mixture containing dGTR. The samples with GTR and dGTR became more rigid; their elongation at break values were lower than those of samples prepared by two-step mixing.

**Figure 6:**
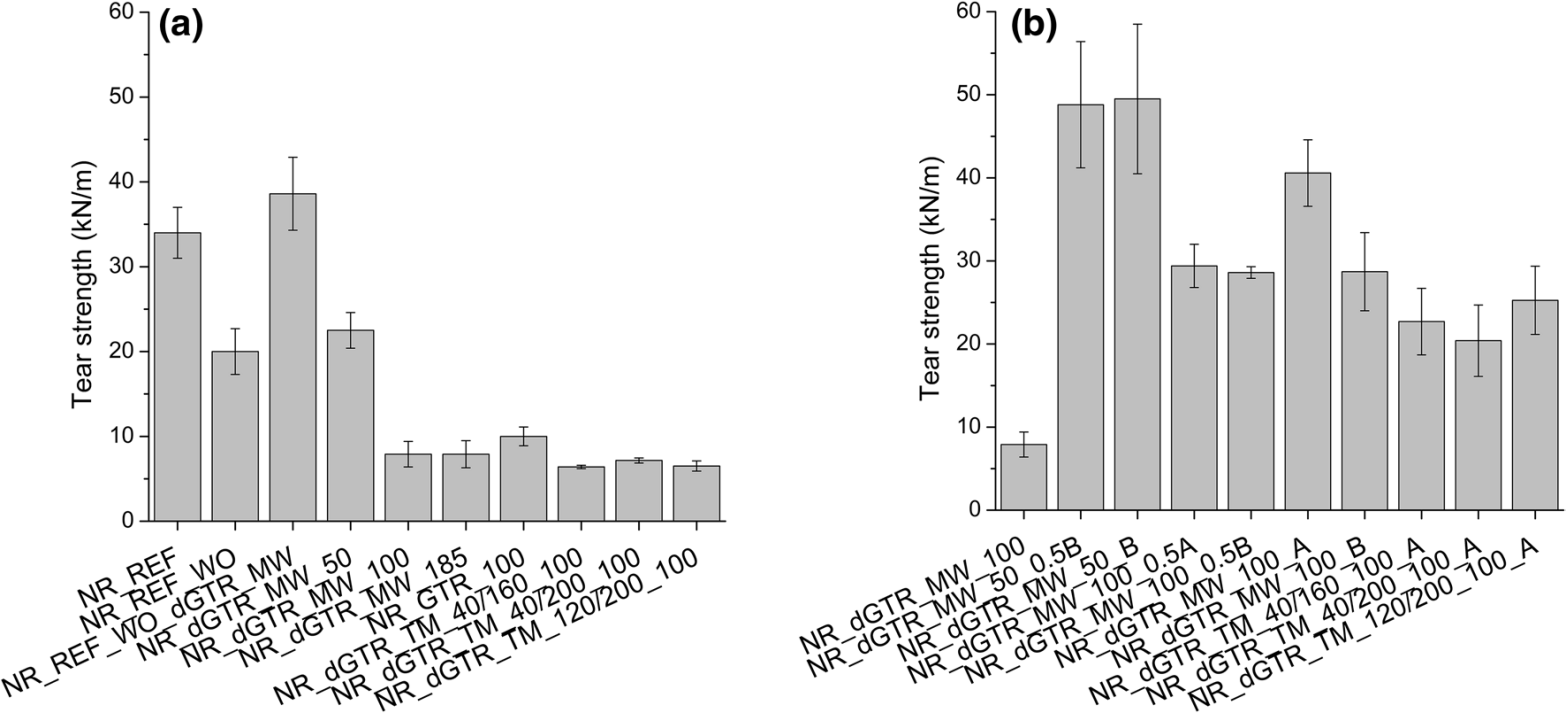
The tear strength of the samples: mixtures containing dGTR (**a**) and containing dGTR and extra vulcanizing agents (**b**).

Tear strength decreased significantly because of the effect of dGTR and GTR (Fig. 6a). However, with additional vulcanizing agents (samples with a code ending with “A” and “B”), tear strength reached and exceeded the values of those of the NR_REF sample (Fig. 6b).

Devulcanized GTR samples suffered degradation, chain scission occurred and the mechanical properties, especially tear strength, dropped significantly when these dGTRs were introduced into NR samples. Extra vulcanization agents helped recover mechanical properties, but the increase was modest because of degraded dGTR particles. However, the shorter and more mobile molecules that formed during microwave devulcanization with the aid of extra vulcanization agents generated more cross-links between the rubber matrix and the surface of the dGTR particles. The improved adhesion between the phases and the different load mode of the tear test caused excellent tear strength in these samples.

**Figure 7:**
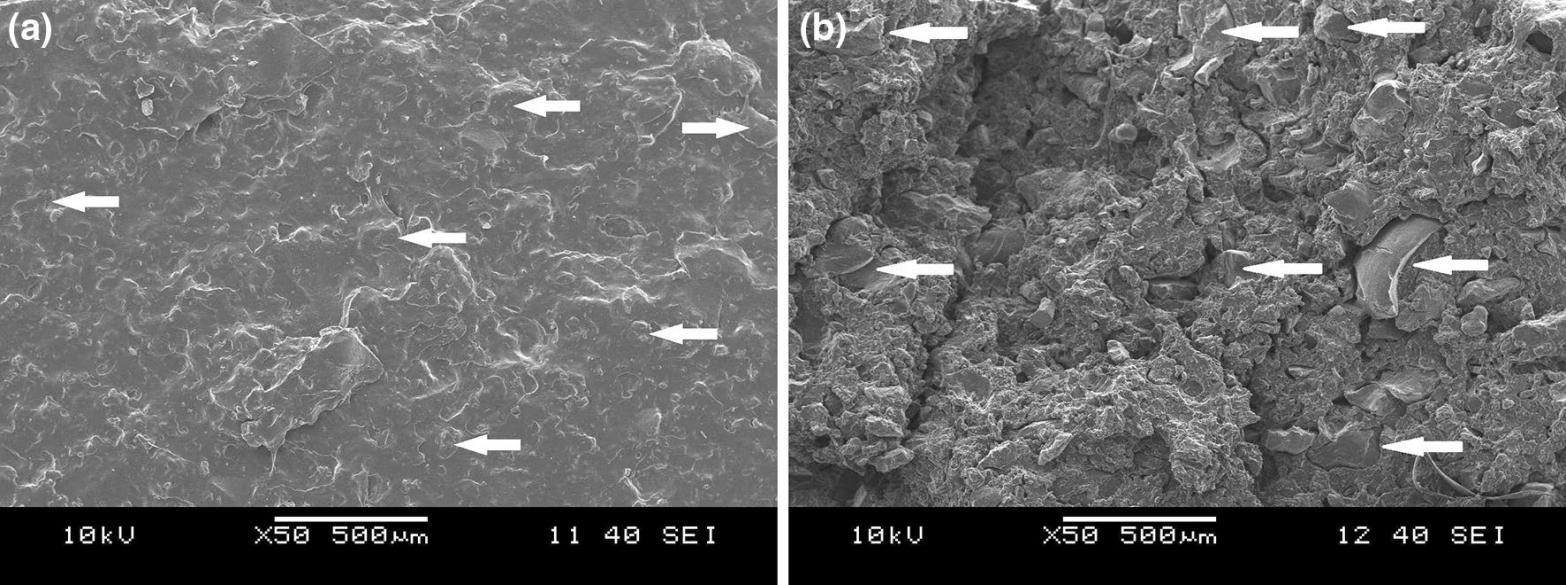
Scanning electron micrograph of the fracture surface of a specimen used for tear strength measurement, (**a**) NR_GTR100 and (**b**) NR_dGTR_MW_100_A sample. Some of the dGTR particles are marked with white arrows.

Figure 7 shows the scanning electron microscopic images of the fracture surface of two tear specimens. Figure 7a shows the relatively smooth tear surface of the NR_GTR100 sample, containing untreated GTR. While, several vertical cracks can be seen in Fig. 7b, indicating the border of dGTR particles (marked with white arrows). It can be inferred that, because of the better adhesion between NR and dGTR (compared to NR and GTR), crack propagation in the dGTR-containing sample required a larger force. In the NR_GTR100 sample, the low interphase adhesion did not allow the GTR component to carry the tensile load. Consequently, GTR particles did not get deformed during the test, hence the smooth appearance of the sample.

## Conclusions

We devulcanized ground tire rubber (GTR) with microwaves in a laboratory oven and thermomechanically in an internal mixer with different rotor speeds and temperatures. Then we characterized the devulcanized GTR (dGTR) samples by Soxhlet extraction and swelling tests to determine their soluble content and cross-link density and performed Horikx’s analysis. In the case of microwave-devulcanized samples, cross-link density was considerably reduced while sol content was high, which suggests that the devulcanization process was dominated by the random degradation of polymer chains. Horikx’s analysis showed that these samples suffered severe degradation. In the case of the thermomechanically devulcanized samples at low temperature and rotor speed settings, the main phenomenon was selective cross-link scission. At higher temperatures and rotor speeds, degradation of the main chains occurred along with cross-link cleavage. Based on Horikx’s analysis, four devulcanized GTR samples were chosen and mixed with NR. dGTR content reduced the tensile strength of the samples drastically, but elongation at break did not follow this trend. Curing curves showed that dGTR has a plasticizing effect on rubber mixtures. The tensile strength of samples containing different dGTRs reflects the results of Horikx’s analysis. The samples containing 100 phr of thermomechanically devulcanised GTR had the same tensile strength as the samples with 50 phr of microwave-devulcanized GTR. Two-step mixing (first adding vulcanization agents to dGTR, then mixing it with the reference rubber mixture) helped recover mechanical properties, especially tear strength.
